# Genetic biomarkers associated with risk and therapeutic response in erectile dysfunction: a systematic review

**DOI:** 10.3389/fphar.2026.1771865

**Published:** 2026-03-24

**Authors:** Letícia Perticarrara Ferezin, Cezar Kayzuka, Mauriely Paiva de Alcântara e Silva, Cecilia Nogueira Tavares Peixeiro, Vitória Carolina Rondon-Pereira, Jose Eduardo Tanus-Santos, Riccardo Lacchini

**Affiliations:** 1 Department of Genetics – Ribeirao Preto Medical School– University of Sao Paulo, Ribeirao Preto, Brazil; 2 Department of Psychiatric Nursing and Human Sciences – Ribeirao Preto College of Nursing – University of Sao Paulo, Ribeirao Preto, Brazil; 3 Department of General and Specialized Nursing - Ribeirao Preto College of Nursing – University of Sao Paulo, Ribeirao Preto, Brazil; 4 Department of Clinical Analyses, Toxicology and Food Science - School of Pharmaceutical Sciences of Ribeirao Preto – University of Sao Paulo, Ribeirao Preto, Brazil; 5 Department of Pharmacology – Ribeirao Preto Medical School – University of Sao Paulo, Ribeirao Preto, Brazil

**Keywords:** biomarkers, erectile dysfunction, genetic polymorphism, personalized medicine, pharmacogenetics

## Abstract

**Introduction:**

Erectile dysfunction (ED) is a multifactorial condition influenced by vascular, neuroendocrine, metabolic, and psychological factors. Growing evidence suggests that genetic variation may contribute to individual susceptibility, severity, and therapeutic response, particularly regarding nitric oxide (NO) signaling and vascular pathways. To systematically synthesize evidence on genetic biomarkers associated with the risk, severity, or therapeutic response of ED in adult men.

**Methods:**

A systematic review was conducted following PRISMA 2020 guidelines and registered in PROSPERO (CRD420251144891). Searches were performed in MEDLINE, Embase, Scopus, LILACS, and Web of Science. Observational studies evaluating genetic polymorphisms or related biomarkers in adult men with Erectile dysfunction were included. Two reviewers independently screened studies, extracted data, and assessed methodological quality using JBI tools and the certainty of evidence using GRADE.

**Results:**

Thirty-five studies met inclusion criteria. The genetic markers most frequently investigated involved pathways related to nitric oxide synthesis and endothelial function, including *NOS3* (eNOS), *NOS1, PDE5A, VEGF, ACE, ARG1/ARG2, DDAH1/2*, and *MTHFR*. Functional variants—particularly *NOS3* G894T, T-786C, and the intron 4 VNTR—were commonly associated with increased ED susceptibility, earlier onset, or greater severity, though results varied across populations. Several polymorphisms influenced pharmacological response to PDE5i, especially variants in *PDE5A, VEGF, eNOS, ARG1/2*, and *DDAH*. However, methodological limitations were pervasive: 77.8% of studies had moderate risk of bias, and 80.6% showed low certainty of evidence. Only one study reached moderate certainty.

**Discussion/Conclusion:**

Current evidence suggests that genetic polymorphisms related to nitric oxide signaling, vascular regulation, and PDE5 inhibitor pharmacodynamics are associated with variability in erectile dysfunction risk and treatment response. However, the overall certainty of evidence is low, and further validation in well-designed, multiethnic studies is required before clinical translation.

## 1 Introduction

Erectile dysfunction (ED) is defined as the persistent inability to achieve or maintain an erection sufficient for satisfactory sexual performance, significantly affecting quality of life and psychosocial wellbeing of both individuals and their partners (Salonia et al., 2025). Clinically, ED may involve vascular, neurogenic, hormonal or psychogenic causes, frequently with multiple factors contributing simultaneously, reflecting its multifactorial nature (MacDonald and Burnett, 2021).

Prevalence estimates of ED vary according to the studied population and diagnostic criteria; in many series, the frequency among men over 40 years exceeds 40%–50% (Lou et al., 2023; Adeyemi et al., 2024). ED is increasingly recognized as a vascular disorder closely linked to aging and impaired cardiometabolic health (Salonia et al., 2025; Dilixiati et al., 2024). The risk factors share common pathophysiological mechanisms involving endothelial dysfunction and impaired nitric oxide signaling, which play a pivotal role in erectile physiology. Although several therapeutic options exist, the introduction of phosphodiesterase type 5 inhibitors (PDE5Is), such as sildenafil, tadalafil and vardenafil, has transformed ED management over the past decades (Adeyemi et al., 2024; Wang et al., 2023). These agents are generally effective and safe, even in patients with cardiovascular comorbidities, but a considerable proportion of patients exhibit suboptimal response, encouraging the search for biological markers of predisposition or therapeutic response (Lacchini and Tanus-Santos, 2014).

In recent years, evidence suggests that genetic variants may influence both susceptibility to ED and variability in treatment outcomes. For instance, our recent study identified that the rs2682826 polymorphism in the *NOS1* gene (nNOS) is associated with more severe erectile dysfunction (Perticarrara Ferezin et al., 2023). Moreover, polymorphisms in the *PDE5A* gene, which encodes the molecular target of PDE5 inhibitors, have been shown to influence the efficacy of sildenafil therapy, particularly among patients with cardiovascular comorbidities (Marchal-Escalona et al., 2016).

Similarly, genetic variants affecting endothelial nitric oxide (NO) signaling have been associated with differences in therapeutic response, supporting the contribution of NO–dependent pathways to interindividual variability in ED (e.g., rs2070744) (Segura et al., 2019). In line with this, systematic reviews suggest that genetic variability across key pathways involved in NO signaling (*NOS1*), vascular function (*NOS3 and PDE5A*), and angiogenesis (*VEGF*) may underlie the heterogeneity in pharmacological outcomes, reinforcing the potential role of pharmacogenetics in ED management (Mostafa et al., 2020).

Despite these advances, current evidence on genetic and molecular biomarkers of ED remains fragmented, heterogeneous, and often limited to small candidate-gene studies, with few attempts to systematically integrate findings across genetic, epigenetic, and pharmacogenetic domains. Furthermore, no comprehensive synthesis has yet clarified which specific genetic biomarkers are consistently associated with ED pathophysiology or therapeutic outcomes, and how these biomarkers may guide the development of personalized management strategies.

### 1.1 Rationale

Given the multifactorial nature of erectile dysfunction and the growing but heterogeneous body of genetic and pharmacogenetic evidence, a systematic and critical synthesis of existing studies is warranted. Current literature lacks an integrated evaluation of genetic biomarkers associated with ED risk, severity, and therapeutic response, particularly across nitric oxide signaling, vascular regulation, and pharmacological pathways. Addressing this gap is essential to clarify the consistency, limitations, and translational relevance of reported genetic associations, as well as to identify priorities for future research.

Therefore, this systematic review was conducted to identify and analyze genetic biomarkers associated with erectile dysfunction and those influencing therapeutic response. By consolidating and critically evaluating the available evidence, this work aims to enhance understanding of the genetic variants involved in ED pathophysiology and to support future advances toward risk stratification and personalized approaches in sexual health. We hypothesized that existing evidence supports associations between genetic polymorphisms involved in nitric oxide signaling, vascular regulation, and drug metabolism pathways and interindividual variability in erectile dysfunction risk and therapeutic response.

## 2 Materials and methods

This systematic review was conducted in accordance with the Preferred Reporting Items for Systematic Reviews and Meta-Analyses (PRISMA) (Rethlefsen and Page, 2022) and followed the methodological recommendations described in the Methods to systematically review and meta-analyze observational studies: a systematic scoping review of recommendations (Mueller et al., 2018). The review process included the formulation of the research question, development of eligibility criteria, construction and execution of search strategies, database searches, screening of titles and abstracts, full-text assessment, evaluation of methodological quality, extraction of relevant data, and narrative synthesis of the findings. The protocol for this review has been previously registered in PROSPERO (ID: CRD420251144891).

### 2.1 Research question

The research question was developed using the PEO structure, which ensured the alignment of the review objective with the eligibility criteria. The guiding question was: “Which genetic biomarkers are associated with the risk, severity, or therapeutic response of erectile dysfunction in adult men with erectile dysfunction?” This structure guided the entire methodological design and study selection process. The details of this approach are presented in Table 1.

**TABLE 1:** PEO framework for research question.

Description	Abbreviation	Components
Population	P	Adult men with erectile dysfunction
Exposure	E	Genetic biomarkers
Outcome	O	Risk, severity, or therapeutic response of erectile dysfunction

Source: Prepared by the author.

### 2.2 Eligibility criteria

Studies were eligible if they included men aged 18–80 years with complaints of sexual dysfunction and if erectile function had been clinically assessed. Eligible studies employed an experimental design that included either a control group or post-pharmacological intervention evaluation. Studies were excluded if they did not account for relevant confounding factors such as hypogonadism, hypothyroidism, spinal cord injury, excessive alcohol consumption, or severe cardiovascular disease. Additional exclusion criteria included the absence of genetic biomarker assessment, evident methodological flaws, use of animal models, or failure to meet Hardy–Weinberg equilibrium. These criteria ensured the inclusion of studies with adequate methodological rigor and relevance to the research question.

### 2.3 Sources of information

The bibliographic search was performed across five electronic databases: MEDLINE/PubMed, Embase, Scopus, LILACS, and Web of Science. Controlled vocabularies (MeSH, DeCS, Emtree) and free-text terms were identified through preliminary searches and were systematically combined using Boolean operators AND and OR. Search strategies were adapted for each database to maximize sensitivity and specificity and are presented in Table 2. All retrieved citations were exported to Rayyan QCRI software for organization and screening.

**TABLE 2:** Search strategies applied in the systematic review for identifying and analyzing genetic biomarkers linked to erectile dysfunction and determinants of therapeutic response, based on the consulted databases.

Database	Controlled terms/free terms
MEDLINE	(((“erectile dysfunction” [MeSH Terms] OR (“erectile” [All Fields] AND “dysfunction” [All Fields]) OR “erectile dysfunction” [All Fields] OR “erectile dysfunction” [MeSH Terms]) AND (“polymorphism, genetic” [MeSH Terms] OR (“polymorphism” [All Fields] AND “genetic” [All Fields]) OR “genetic polymorphism” [All Fields] OR “polymorphism genetic” [All Fields])) OR “polymorphism, genetic” [MeSH Terms] OR (“pharmacogenetically” [All Fields] OR “pharmacogenetics” [MeSH Terms] OR “pharmacogenetics” [All Fields] OR “pharmacogenetic” [All Fields]) OR “pharmacogenetics” [MeSH Terms] OR (“pharmacogenetics” [MeSH Terms] OR “pharmacogenetics” [All Fields] OR “pharmacogenomic” [All Fields] OR “pharmacogenomics” [All Fields] OR “pharmacogenomically” [All Fields]) OR “pharmacogenetics” [MeSH Terms] OR (“biomarker s” [All Fields] OR “biomarkers” [Supplementary Concept] OR “biomarkers” [All Fields] OR “biomarker” [All Fields] OR “biomarkers” [MeSH Terms]) OR “biomarkers” [MeSH Terms]) AND ((excludepreprints [Filter]) AND (classicalarticle [Filter] OR clinicalstudy [Filter] OR clinicaltrial [Filter] OR clinicaltrialprotocol [Filter] OR clinicaltrialphasei [Filter] OR clinicaltrialphaseii [Filter] OR clinicaltrialphaseiii [Filter] OR clinicaltrialphaseiv [Filter] OR comparativestudy [Filter] OR controlledclinicaltrial [Filter] OR multicenterstudy [Filter] OR observationalstudy [Filter] OR pragmaticclinicaltrial [Filter] OR randomizedcontrolledtrial [Filter]) AND (humans [Filter]) AND (male [Filter]) AND (english [Filter] OR portuguese [Filter] OR spanish [Filter]) AND (alladult [Filter] OR youngadult [Filter] OR adult [Filter] OR middleagedaged [Filter] OR middleaged [Filter] OR aged [Filter] OR 80andover [Filter])
LILACS	(mh: (disfunção erétil)) OR (disfunção erétil) OR (mh: (impotência sexual)) OR (impotência sexual) OR (mh: (erectile dysfunction)) OR (erectile dysfunction) AND (mh: (polimorfismo genético)) OR (polimorfismo genético) OR (mh: (variante genética)) OR (variante genética) OR (mh: (genetic polymorphism)) OR (genetic polymorphism) OR (mh: (farmacogenética)) OR (farmacogenética) OR (mh: (farmacogenômica)) OR (farmacogenômica) OR (mh: (pharmacogenetics)) OR (pharmacogenetics) OR (mh: (pharmacogenomics)) OR (pharmacogenomics) OR (mh: (biomarcadores)) OR (biomarcadores) OR (mh: (marcadores biológicos)) OR (marcadores biológicos) OR (mh: (biomarkers)) OR (biomarkers) AND type_of_study: (“risk_factors_studies” OR “observational_studies” OR “etiology_studies” OR “clinical_trials” OR “diagnostic_studies” OR “prevalence_studies” OR “prognostic_studies” OR “qualitative_research” OR “incidence_studies” OR “overview”) AND instance:“regional”
Embase	(‘erectile dysfunction’/exp OR ‘erectile dysfunction’) AND (‘genetic polymorphism’/exp OR ‘genetic polymorphism’ OR ‘pharmacogenetics’/exp OR ‘pharmacogenetics’ OR ‘pharmacogenomics’/exp OR ‘pharmacogenomics’ OR ‘biomarker’/exp OR biomarker) AND (‘case control study’/de OR ‘clinical article’/de OR ‘clinical protocol’/de OR ‘clinical study’/de OR ‘clinical trial’/de OR ‘clinical trial topic’/de OR ‘cohort analysis’/de OR ‘comparative effectiveness’/de OR ‘comparative study’/de OR ‘control group’/de OR ‘controlled clinical trial’/de OR ‘controlled clinical trial topic’/de OR ‘controlled study’/de OR ‘correlational study’/de OR ‘cross sectional study’/de OR ‘diagnostic test accuracy study’/de OR ‘drug dosage form comparison’/de OR ‘drug dose comparison’/de OR ‘evidence based medicine’/de OR ‘good clinical practice’/de OR ‘human cell’/de OR ‘human experiment’/de OR ‘human tissue’/de OR ‘intervention study’/de OR ‘linear regression analysis’/de OR ‘logistic regression analysis’/de OR ‘longitudinal study’/de OR ‘major clinical study’/de OR ‘multicenter study’/de OR ‘multiple linear regression analysis’/de OR ‘multivariate logistic regression analysis’/de OR ‘observational study’/de OR ‘phase 1 clinical trial’/de OR ‘phase 1 clinical trial topic’/de OR ‘phase 2 clinical trial’/de OR ‘phase 2 clinical trial topic’/de OR ‘phase 3 clinical trial’/de OR ‘phase 3 clinical trial topic’/de OR ‘phase 4 clinical trial’/de OR ‘population based case control study’/de OR ‘prospective study’/de OR ‘qualitative research’/de OR ‘quality control’/de OR ‘randomized controlled trial’/de OR ‘randomized controlled trial topic’/de OR ‘regression analysis’/de OR ‘retrospective study’/de OR ‘sample size’/de OR ‘secondary analysis’/de OR ‘statistical model’/de OR ‘theoretical study’/de OR ‘validation study’/de) AND ([adult]/lim OR [aged]/lim OR [middle aged]/lim OR [very elderly]/lim OR [young adult]/lim) AND [male]/lim
Scopus	((TITLE-ABS-KEY (erectile dysfunction) AND TITLE-ABS-KEY (genetic polymorphism) OR TITLE-ABS-KEY (pharmacogenetics) OR TITLE-ABS-KEY (pharmacogenomics) OR TITLE-ABS-KEY (biomarker)) AND (LIMIT-TO (DOCTYPE,”ar”)))
Web of Science	erectile dysfunction (Topic) and genetic polymorphism (Topic) and genetic polymorphism (Abstract) or pharmacogenetics (Topic) and pharmacogenetics (Abstract) or pharmacogenomics (Topic) and pharmacogenomics (Abstract) or biomarker (Topic) and biomarker (Abstract) and men (Topic) and Article (Document Types) and Article (Document Types) and English or Spanish or Portuguese (Languages) and Oncology or Biochemistry Molecular Biology or Neurosciences or Medicine Research Experimental or Multidisciplinary Sciences or Medicine General Internal or Clinical Neurology or Chemistry Analytical or Pharmacology Pharmacy or Immunology or Cell Biology or Orthopedics or Allergy or Developmental Biology or Transplantation or Behavioral Sciences or Psychology or Virology or Primary Healthcare or Imaging Science Photographic Technology or Physics Atomic Molecular Chemical or Genetics Heredity or Biotechnology Applied Microbiology or Endocrinology Metabolism or Cardiac Cardiovascular Systems or Radiology Nuclear Medicine Medical Imaging or Nanoscience Nanotechnology or Psychiatry or Biochemical Research Methods or Otorhinolaryngology or Sport Sciences or Computer Science Artificial Intelligence or Anesthesiology or Audiology Speech Language Pathology or Computer Science Software Engineering or Gastroenterology Hepatology or Surgery or Pathology or Toxicology or Respiratory System or Geriatrics Gerontology or Materials Science Multidisciplinary or Urology Nephrology or Biology or Microbiology or Electrochemistry or Gerontology or Emergency Medicine or Integrative Complementary Medicine or Parasitology or Substance Abuse or Rehabilitation or Education Special or Psychology Applied or Psychology Social or Nutrition Dietetics or Psychology Clinical or Tropical Medicine or Chemistry Organic or Andrology or Health Policy Services or Nursing or Family Studies or Sociology or Infectious Diseases or Mathematical Computational Biology or Engineering Biomedical or Biophysics or Hematology or Education Educational Research or Medical Ethics or Social Work or Medicine Legal or Psychology Multidisciplinary or Psychology Experimental or Psychology Developmental or Peripheral Vascular Disease or Rheumatology or Physics Applied or Ophthalmology or Physiology or Food Science Technology or Healthcare Sciences Services or Chemistry Medicinal or Chemistry Physical or Social Issues or Social Sciences Biomedical or Meteorology Atmospheric Sciences or Critical Care Medicine or Geosciences Multidisciplinary or Neuroimaging or Dentistry Oral Surgery Medicine or Reproductive Biology or Dermatology or Psychology Educational (Web of Science Categories) and All Open Access (Open Access)

Source: Prepared by the author.

### 2.4 Screening process

**FIGURE 1:**
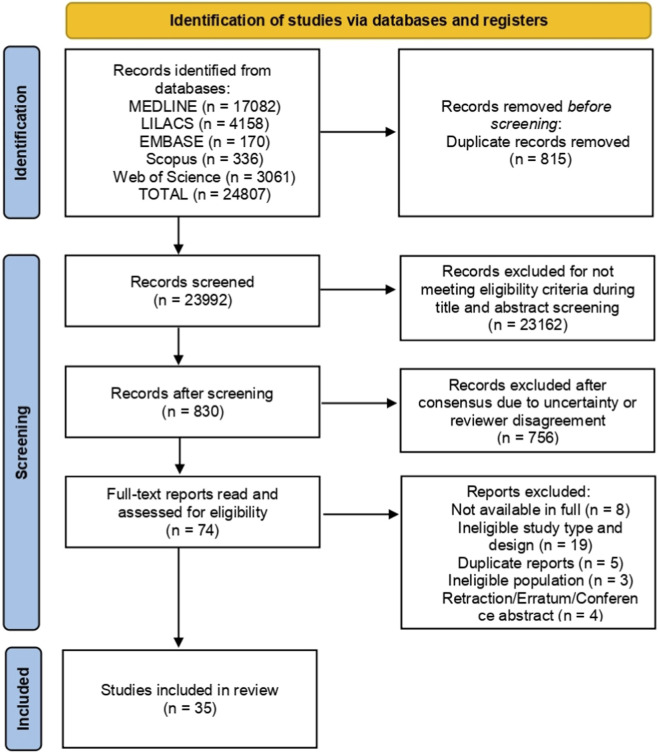
PRISMA flowchart of study selection in the systematic review on genetic biomarkers associated with the risk, severity, or therapeutic response of erectile dysfunction in adult men.

All references obtained from the databases were imported into Rayyan QCRI, where duplicate entries were removed. Two independent reviewers then screened titles and abstracts based on predefined inclusion and exclusion criteria. Articles that met the criteria or presented insufficient information were retrieved for full-text analysis. The same reviewers independently assessed the full texts to determine final eligibility. Discrepancies at any stage were resolved through discussion or consultation with a third reviewer. The full selection process, including numbers of identified, screened, excluded, and included records, was documented through a PRISMA 2020 flow diagram (Figure 1).

### 2.5 Data extraction process

Data extraction was performed independently by two reviewers using a standardized form developed according to the Joanna Briggs Institute guidelines for systematic reviews of etiology and risk. Extracted data included authors, journal, study objectives, design, setting, sample characteristics, recruitment procedures, follow-up duration, exposure variables (genetic biomarkers), outcome variables, analytic methods, adjustment for confounding factors, and main results. Any discrepancies between reviewers were resolved through discussion or involvement of a third reviewer. When necessary, study authors were contacted to obtain missing or clarifying information. Extracted data were organized to allow structured comparison across studies. When available, measures of effect size (e.g., odds ratios or relative risks) and corresponding confidence intervals were extracted. However, due to heterogeneity in study designs, statistical models, and reporting practices, effect size estimates were not consistently available and therefore could not be uniformly presented.

### 2.6 Assessment of the quality of studies

The methodological quality of the included studies was evaluated through a two-step approach. First, the risk of bias was assessed using the Joanna Briggs Institute (JBI) (Moola et al., 2015) critical appraisal instruments specific to each study design. These tools examine key methodological domains, including the clarity of inclusion criteria, the adequacy of participant and setting descriptions, the validity and reliability of exposure and outcome measurements, the identification and management of potential confounding factors, and the appropriateness of the statistical analyses. Each item was rated as “yes,” “no,” “unclear,” or “not applicable,” with “yes” responses scored as one point and all other responses scored as zero. The overall JBI score for each study was calculated as the proportion of criteria met, allowing the classification of the study’s methodological rigor.

Secondly, the level of evidence and the certainty of the body of evidence were assessed using the GRADE (Grades of Recommendation, Assessment, Development and Evaluation) (Colunga-Lozano et al., 2025) framework. This system considers factors such as study limitations (risk of bias), inconsistency, indirectness, imprecision, and publication bias to classify the certainty of evidence as high, moderate, low, or very low. Together, the JBI and GRADE assessments provided a comprehensive evaluation of both the internal validity of each study and the overall confidence in the synthesized findings. These sources of heterogeneity further limited the feasibility of quantitative meta-analysis.

### 2.7 Summary of results

Given the heterogeneity of study designs, populations, biomarkers evaluated, and analytical methods, the results were synthesized narratively. Specifically, substantial variability in ancestry, phenotypic definitions of erectile dysfunction, genetic variants assessed, outcome measures, and reporting of effect sizes precluded formal quantitative synthesis and meta-analysis. Therefore, a narrative synthesis was adopted to contextualize findings within their respective methodological and population-specific frameworks and to avoid inappropriate pooling of heterogeneous data.

## 3 Results

Through the search conducted across the selected databases, a total of 24,807 records were identified. After removing 815 duplicate entries, 23,992 studies were screened based on titles and abstracts. Of these, 23,162 studies were excluded for not meeting the eligibility criteria. A total of 830 reports were sought for full-text retrieval, of which 74 full-text articles were assessed for eligibility. Following this assessment, 39 studies were excluded for reasons such as unavailability of the full text, language incompatibility, ineligible study design, ineligible population, retraction, or insufficient information for confirmation based on the abstract. Ultimately, 35 studies investigating genetic biomarkers associated with the risk, severity, or therapeutic response of erectile dysfunction in adult men were included in the review. Figure 1 presents the detailed flowchart of the study selection process. The list of full-text articles excluded after eligibility assessment, along with the corresponding reasons for exclusion, is provided in Supplementary Table S1.

The studies included in this systematic review comprised different observational designs, including cross-sectional, case–control, and prospective cohort studies. The study populations and main findings are summarized in Table 3.

**TABLE 3:** Characteristics and main findings of published studies investigating genetic biomarkers associated with the risk, severity, or therapeutic response of erectile dysfunction.

Reference	Journal	Study design	Population/Country of study	Main results
García-Cardoso et al. (2024)	Journal of Personalized Medicine	Prospective cohort study	Non-diabetic men with normal baseline erectile function undergoing nerve-sparing radical prostatectomy	The *PDE5A* rs3806808 polymorphism showed no association with PDE5 activity or therapeutic response
Pallotti et al. (2024)	Journal of Endocrinological Investigation	Cross-sectional study	Sexually active men aged 18–65 years with a prior diagnosis of acromegaly, all with controlled GH/IGF-1 levels; most were post–pituitary adenoma surgery	Erectile dysfunction was not associated with the *AR* (CAG) or *ERβ* (CA) polymorphisms
Zhuravleva et al. (2021)	The Journal of Sexual Medicine	Cross-sectional study	Young Finnish men from the general population, including monozygotic and dizygotic twins and siblings; extensive hormonal and genetic assessment; low prevalence of moderate/severe ED; no specific ED therapy	None of the *AR, SHBG*, or *SRD5A2* polymorphisms showed significant association with ED in young men
Rocca et al. (2020)	Frontiers in Pharmacology	Prospective observational study	Men with new-onset erectile dysfunction, low/intermediate cardiovascular risk, no major systemic diseases, and no severe hypogonadism or hepatic/renal failure	SNPs in *PDE11A* and *CYP2D7* may predict therapeutic efficacy, while SNPs in *PDE2A, PDE3A*, and *CYP2C9* may predict adverse drug reactions. Multi-omics integration may enhance therapeutic personalization
Arda et al. (2019)	Systems Biology in Reproductive Medicine	Prospective case-control study	Turkish men; controls without ED; cases with confirmed vasculogenic ED; higher rates of hypertension, diabetes, CAD, and smoking among cases (all p < 0.001)	The *NOS3* intron 4 VNTR (a/a) genotype was significantly associated with vasculogenic ED and disease severity. *NOS3* rs1799983 did not distinguish cases from controls but may relate to ED severity
Brites-Anselmi et al. (2019)	Nitric Oxide	Case-control study	Men with well-characterized vasculogenic ED; excluded psychogenic, endocrine, neurological, anatomical, post-prostatectomy, and traumatic causes; diabetics included only in the ED group	Polymorphisms in *DDAH1* and *DDAH2* modulated ADMA and nitrite levels only in men with ED, but did not increase ED risk or severity
Segura et al. (2019)	Gene: X	Prospective observational study	Spanish men aged 18–80 years undergoing long-term opioid therapy; no prior use of testosterone or PDE5 inhibitors; no substance dependence; no ED before pain onset; no severe comorbidities	The *NOS3* rs2070744 influenced both baseline ED severity and therapeutic response in men with chronic pain; individuals with the CC genotype showed the greatest improvement despite worse baseline function. rs1799983 and 4VNTR showed no significant associations
Mostaza et al. (2018)	Journal of Clinical Lipidology	Population-based cross-sectional study	Adults aged 45–74 years without known cardiovascular disease; low frequency of the T allele (2.9%); Spanish ethnicity	The *PCSK9* rs11591147 variant was associated with lower ED prevalence and reduced carotid intima-media thickness, suggesting vascular protection independent of traditional risk factors
Lacchini et al. (2018)	The Pharmacogenomics Journal	Prospective observational study	Men with ED treated in a urology outpatient clinic; two groups: post-prostatectomy and clinically vasculogenic ED.	Polymorphisms in *ARG1* and *ARG2* influenced arginase activity and modulated sildenafil response, supporting their potential as pharmacogenetic biomarkers
Azevedo et al. (2017)	Nitric Oxide	Prospective observational cohort	Men with ED seen in a urology outpatient clinic; subgroups: CED (mainly vasculogenic) and PPED (post-radical prostatectomy); diabetics included with various cardiovascular comorbidities	Plasma ADMA and nitrite levels may reflect sildenafil responsiveness in CED; *DDAH1* variants relate to ADMA levels; *DDAH2* rs805304/rs805305 may predict better sildenafil response in PPED.
Yang et al. (2017)	The Journal of Sexual Medicine	Case–control	Han Chinese men; ED defined by IIEF-5 <21 with normal testosterone; excluded psychogenic ED, pelvic/penile surgery, and drug abuse; age-matched healthy controls	Functional *NOS3* polymorphisms influence susceptibility, age of onset, and severity of ED in Han Chinese men, but do not affect sildenafil response
Lee et al. (2017)	The Journal of Sexual Medicine	Cross-sectional, population-based	Taiwanese men >40 years from a health-screening program; comprehensive clinical and lab assessment; excluded prior PDE5i use, hypogonadism, anatomic abnormalities, neurologic disease, malignancy, psychiatric disorders, or substance abuse	The *VEGF* rs699947 A allele is associated with higher prevalence and greater severity of ED, suggesting a genetic susceptibility marker
Marchal-Escalona et al. (2016)	The Journal of Sexual Medicine	Prospective cohort	Caucasian men with coronary artery disease post-angioplasty, classified as low cardiac risk; high use of beta-blockers (82%), statins (85%), and oral antidiabetics (40%); no nitrate therapy; high prevalence of metabolic syndrome	Sildenafil response depends on *PDE5A* polymorphisms—especially rs3806808—and interacts with glycemic status; diabetic carriers of the G allele show poorer response
Liu et al. (2015)	Andrology	Cross-sectional	Taiwanese men ≥40 years in a health-screening program, sexually active ≥6 months; extensive clinical and lab evaluation; high rates of DM (10.7%), hypertension (30.1%), hyperlipidemia (20.9%), LUTS (49%), and smoking (14.6%)	ED risk depends on the interaction between testosterone and *AR* CAG length: with normal TT (>330 ng/dL), longer CAG increases ED risk; with low TT, CAG length has no major effect
Lacchini et al. (2013)	The Pharmacogenomics Journal	Prospective observational	Brazilian men with ED from a university clinic; excluded endocrine, psychiatric, neurogenic, or anatomic causes, improper sildenafil use, and prostatectomy <1 year; CED subgroup enriched for vasculogenic ED.	*VEGF* polymorphisms strongly modulate sildenafil response in both PED and CED; rs1570360 AA genotype and rs699947 A allele predict poorer response due to reduced VEGF expression and impaired NO–cGMP signaling
Muniz et al. (2013)	The Pharmacogenomics Journal	Prospective observational	Brazilian men aged 40–80 years with documented ED; pre/post sildenafil assessment; excluded endocrine, psychiatric, neurogenic causes, hypogonadism, penile implants, stroke, CNS trauma, Peyronie’s disease, and prostatectomy <1 year	PED: rs2070744 (C allele) and haplotype H6 enhance sildenafil responsiveness; CED: intron-4 VNTR (4a allele) improves response; *NOS3* variants differentially affect sildenafil pharmacodynamics by ED etiology
Hermans et al. (2012)	European Journal of Clinical Investigation	Cross-sectional	Adults with type 2 diabetes, mostly Caucasian (90%); long disease duration (19 ± 9 years) and high prevalence of metabolic syndrome, hypertension, microangiopathy, and macroangiopathy; ED analysis performed only in men	In T2DM men, the *NOS3* rs1799983 TT genotype is strongly linked to higher ED prevalence and is protective against ocular hypertension/glaucoma; not associated with micro/macrovascular complications or glycemic control
Safarinejad et al. (2011)	BJU International	Case–control	Men with vasculogenic ED confirmed by penile Doppler after PGE1; strictly excluded diabetes, hypertension, hypercholesterolemia, obesity, smoking, heavy alcohol use, cardiovascular, endocrine, neurologic, or psychiatric diseases, and ED-related medications; healthy controls without comorbidities	*NOS3* rs2070744 and rs1799983 are strong genetic predictors of vasculogenic ED, independent of clinical risk factors; intron 4 4a/4b shows no association; combined rs2070744 C + rs1799983 T alleles further increase risk
Andersen et al. (2011)	Fertility and Sterility	Cross-sectional, population-based	Population-based EPISONO sample (São Paulo); broad age range (20–80 years); questionnaire, polysomnography, labs, hormones, and genotyping; ED associated with older age, higher BMI, diabetes, hypertension, and AHI >15	The PROGINS polymorphism is not associated with ED and does not influence hormone levels; other PR variants may still warrant investigation
Safarinejad et al. (2010)	Archives of Medical Research	Case–control	Men aged 22–40 years; NIH-defined ED with vasculogenic origin confirmed by duplex Doppler after PGE1; excluded psychogenic, neurogenic, endocrine causes, heavy smoking, alcohol use, major comorbidities, and ED-inducing drugs	*MTHFR* rs1801133 is a strong risk marker for early-onset vasculogenic ED, partly mediated by hyperhomocysteinemia; rs1801131 and rs2274976 affect Hcy but do not independently increase risk; T-allele genotype combinations and haplotypes markedly elevate susceptibility
Sinici et al. (2010)	Urology	Case-control study	Turkish population with comprehensive clinical evaluation, vascular Doppler, and comorbidity assessment; diabetes and hypertension more prevalent in cases; obesity and dyslipidemia not significantly different	The CC genotype of *NOS3* rs2070744 was an independent risk factor for ED; intron 4 VNTR did not influence risk
Lombardo et al. (2010)	Journal of Sexual Medicine	Prospective cohort study	Men with non-anatomical ED; excluded age >66 years, obesity, diabetes, metabolic syndrome, hypogonadism, hormonal disorders, cardiovascular family history, and ED-inducing medications; comprehensive evaluation with IIEF, hormonal tests, lipids, glucose, SHBG, and alprostadil-induced Doppler	The TT genotype (*MTHFR* rs1801133), linked to hyperhomocysteinemia and low folate, increased risk of vascular ED and sildenafil non-response. Folate + vitamin B6 supplementation improved erectile function and reduced homocysteine
Andersen et al. (2010b)	Journal of Sexual Medicine	Cross-sectional study	Men from São Paulo evaluated in the EPISONO population-based study with hormonal testing, polysomnography, assessment of hypertension, diabetes, BMI, and genetic ancestry (European, African, Amerindian)	The *NOS3* rs1799983 polymorphism showed no association with ED in the Brazilian population; age was the only independent predictor
Eisenhardt et al. (2010)	Andrologia	Case-control study	Men with high burden of vascular comorbidities: 72 with diabetes, 109 with hypertension, 21 with coronary disease, and 9 with dyslipidemia; ED of mixed etiologies (vasculogenic, neurogenic, psychogenic, hormonal, or combined)	Polymorphisms *GNB3* rs5443, ACE I/D, and *NOS3* rs1799983 did not influence ED risk, age of onset, or vascular response
Andersen et al. (2010a)	Journal of Sexual Medicine	General population cross-sectional study	Participants of the EPISONO epidemiological study, evaluated through structured interviews, polysomnography, and laboratory tests	Although ACE I/D was not associated with ED in the general population, the I allele increased risk among men aged 40–55, suggesting age-dependent effects
Meluzín et al. (2009)	Heart and Vessels	Cross-sectional/clinical cohort study	Patients with suspected or confirmed coronary artery disease, with high prevalence of risk factors: hypertension (74%), hypercholesterolemia (64%), diabetes (25%), smoking (63%), and obesity (30%)	ED was highly prevalent and correlated with greater extent and severity of coronary artery disease; no *NOS3* polymorphism associated with ED. ED combined with diabetes was a strong marker of severe CAD.
Erol et al. (2009)	Journal of Sexual Medicine	Case-control study	Men with organic or mixed ED; excluded pure psychogenic ED; controls without ED but with similar vascular risk profiles; complete clinical evaluation including metabolic labs and Doppler when indicated	The T allele of *NOS3* rs1799983 increased ED risk; the intron 4 VNTR polymorphism showed no association
Lee et al. (2009)	Journal of Sexual Medicine	Analytical cross-sectional study	Men ≥45 years undergoing health screening; sexually active; excluded those with prostate cancer, PSA >4 ng/mL, prior urological surgery, psychiatric disorders, use of ED or LUTS medications, or neurological disease	The T allele of *NOS3* rs1799983 was an independent factor for ED and BPH/LUTS; carriers exhibited worse erectile function
Mazo et al. (2008)	International Journal of Impotence Research	Analytical cross-sectional study	Men with metabolic syndrome defined by NCEP/ATP III criteria	The DD genotype of *ACE* I/D was associated with increased ED risk and severity in men with metabolic syndrome; the D allele may reflect macro- and microvascular vulnerability
Lee et al. (2008)	Journal of Sexual Medicine	Case-control study	Sexually active Taiwanese men with regular partners; excluded hypoactive sexual desire, penile abnormalities, spinal cord injury, prostatectomy, psychiatric disorders, or substance abuse; complete clinical, psychological, laboratory, and hormonal evaluation	The *GNB3* rs5443 polymorphism was not associated with ED prevalence, but the T allele correlated with poorer erectile scores and higher prevalence of metabolic comorbidities (diabetes, hypertension, elevated BMI)
Lee et al. (2007)	BJU International	Non-consecutive clinical cohort study	Sexually active Taiwanese men with regular partners; same exclusion criteria as above; complete sexual, physical, laboratory, and hormonal assessment	Diabetes, age, and hypogonadism were independent predictors of ED; the T allele (*NOS3* rs1799983) increased ED risk and severity
Peskircioglu et al. (2007)	International Journal of Impotence Research	Case-control study	Men with ED of multiple etiologies (vasculogenic, psychogenic, endocrine, mixed, neurological); evaluated with full clinical history, physical exam, IIEF-5, lab tests, hormones, and Doppler when indicated; excluded those with contraindications to sildenafil	The intron 4 VNTR variant of *NOS3* influenced sildenafil response (allele a → better response), while rs1799983 and rs3918226 showed no effect on ED risk or therapeutic response
Eisenhardt et al. (2003)	Urology	Analytical cross-sectional study	Men with ED of various etiologies evaluated according to European Urology Association guidelines; 44% had cardiovascular comorbidities (diabetes, hypertension, angina, hypercholesterolemia)	Polymorphisms *ACE* I/D and *NOS3* rs1799983 influenced sildenafil response (*ACE* II: better response; *NOS3* TT: poorer response), especially in men with cardiovascular disease
Liu et al. (2024)	American Journal of Men’s Health	Analytical cross-sectional study	Taiwanese men aged ≥40 years participating in a screening program with complete urological, metabolic, and genetic assessment	The A allele (*VEGF* rs699947) was associated with increased ED risk, poorer erectile function, and higher prevalence of metabolic syndrome, suggesting a shared genetic pathway
Perticarrara Ferezin et al. (2023)	Life	Case-control, observational, cross-sectional study	233 adult men (119 with erectile dysfunction diagnosed by IIEF <22 and 114 healthy controls without ED, IIEF ≥22), recruited from a university hospital urology clinic and the community	No association between *NOS1* or *PDE5A* polymorphisms and risk of ED. The *NOS1* rs2682826 CT/TT genotypes were associated with more severe ED. No associations found with plasma nitrite levels

Source: Prepared by the author.

The studies included in this review examined a broad spectrum of genetic biomarkers related to ED, encompassing single nucleotide polymorphisms (SNPs), variable number tandem repeats (VNTRs), insertion/deletion variants (indels), promoter variants, gene-specific repetitive elements, and multivariant haplotype structures. Table 4 provides an organized overview of the genetic markers assessed in each study, specifying both the type of biomarker and the exact gene/variant investigated.

**TABLE 4:** Genetic biomarkers analyzed in the studies included in the systematic review.

Reference	Type of genetic biomarker	Gene/Variant
García-Cardoso et al. (2024)	SNP	PDE5A (rs3806808)
Pallotti et al. (2024)	SNPs	AR (CAG microsatellite); ERβ (CA microsatellite)
Zhuravleva et al. (2021)	SNPs	AR (CAG microsatellite); AR (rs6152); SRD5A2 (rs2208532/rs523349); SHBG (rs1799941/rs6259)
Rocca et al. (2020)	SNPs detected by NGS	SNPs in 22 target genes (NGS)
Arda et al. (2019)	VNTR + SNPs	NOS3 (Intron 4 variable number of tandem repeats (VNTR)/rs1799983)
Brites-Anselmi et al. (2019)	NPs + haplotypes	DDAH1 (rs1554597/rs18582); DDAH2 (rs805304/rs805305)
Segura et al. (2019)	SNPs + VNTR	NOS3 (rs2070744/rs1799983/Intron 4 VNTR)
Mostaza et al. (2018)	Missense SN	PCSK9 rs11591147
Lacchini et al. (2018)	SNPs + plasma biomarkers	ARG1 and ARG2 (multiple SNPs)
Azevedo et al. (2017)	SNPs	DDAH1 (rs1554597/rs18582); DDAH2 (rs805304/rs805305); and plus plasma ADMA and nitrite levels
Yang et al. (2017)	SNPs and VNTR	NOS3 (rs1799983/rs2070744/intron 4 VNTR)
Lee et al. (2017)	SNPs	VEGF (rs833061/rs1570360/rs699947)
Marchal-Escalona et al. (2016)	SNPs	PDE5A (rs12646525/rs3806808)
Liu et al. (2015)	Repetitive polymorphism	AR (CAG microsatellite)
Lacchini et al. (2013)	SNPs	VEGF (rs699947/rs1570360/rs2010963)
Muniz et al. (2013)	SNP, VNTR, and haplotypes	NOS3 (rs2070744/intron-4 VNTR/rs1799983)
Hermans et al. (2012)	SNP	NOS3 (rs1799983)
Safarinejad et al. (2011)	SNP and VNTR	NOS3 (rs2070744/rs1799983/intron-4 VNTR)
Andersen et al. (2011)	PROGINS Alu insertion	Progesterone receptor (PGR) PROGINS (Alu insertion); T1 (185 bp, wild-type); T2 (485 bp, Alu insertion)
Safarinejad et al. (2010)	SNPs	MTHFR (rs1801133/rs1801131/rs2274976)
Sinici et al. (2010)	SNP + VNTR	NOS3 (rs1799983)
Lombardo et al. (2010)	SNP	MTHFR (rs1801133)
Andersen et al. (2010b)	SNP	NOS3 (rs1799983)
Eisenhardt et al. (2010)	SNP + indel	GNB3 (rs5443); ACE I/D polymorphism (intron 16); NOS3 (rs1799983)
Andersen et al. (2010a)	Indel polymorphism	ACE I/D polymorphism (intron 16)
Meluzín et al. (2009)	SNP + VNTR	NOS3 (rs1799983)
Erol et al. (2009)	SNP + VNTR	NOS3 (rs1799983; Intron 4 VNTR)
Lee et al. (2009)	SNP	NOS3 (rs1799983)
Mazo et al. (2008)	I/D polymorphism	ACE I/D polymorphism (intron 16)
Lee et al. (2008)	SNP	GNB3 (rs5443)
Lee et al. (2007)	SNP	NOS3 (rs1799983)
Peskircioglu et al. (2007)	SNP + VNTR	NOS3 (rs1799983/intron 4 VNTR/IVS23 + 10 G/T)
Eisenhardt et al. (2003)	SNP + I/D	ACE I/D polymorphism (intron 16); NOS3 (rs1799983)
Liu et al. (2024)	SNP	VEGF (rs699947)
Perticarrara Ferezin et al. (2023)	SNPs	NOS1 (rs41279104/rs2682826); PDE5A (rs2389866/rs3733526/rs13124532)

Source: Prepared by the author.

Across the included literature, the most frequently evaluated genes were NOS3 (eNOS), NOS1 (nNOS), ACE, GNB3, VEGF, PCSK9, PDE5A, and genes associated with nitric oxide synthesis, endothelial function, and vascular homeostasis. Collectively, the evidence highlights substantial heterogeneity in the genetic targets explored, with a marked predominance of SNP-based analyses and recurrent emphasis on pathways regulating NO bioavailability, vasodilation, and penile vascular integrity.

The methodological quality of the 36 included studies, assessed with the Joanna Briggs Institute (JBI) tools, was generally suboptimal and heterogeneous. Most studies were rated as having a moderate risk of bias (n = 28; 77.8%), reflecting limitations typical of observational genetic studies, such as incomplete adjustment for confounders, absence or inadequate reporting of Hardy–Weinberg equilibrium, moderate sample sizes, and lack of independent replication. A smaller subset of studies achieved a low risk of bias (n = 5; 13.9%), usually characterized by clearer population definitions, more rigorous control of confounding factors, and more robust outcome assessment. Only three studies (8.3%) were judged to be at high risk of bias, mainly due to more pronounced methodological shortcomings, including poorly defined study populations, possible selection bias, or insufficiently validated outcome measures.

Consistently, the certainty of evidence rated with the GRADE approach was predominantly limited (Table 5). The majority of studies provided low-certainty evidence (n = 29; 80.6%), largely because of observational design, imprecision, and inconsistency in reported genetic associations. An additional group showed very low certainty (n = 6; 16.7%), indicating substantial concerns regarding bias, inconsistency, and lack of reproducibility. Only one study (2.8%) reached a moderate level of certainty, supported by stronger methodological rigor, better control of confounding factors, and more consistent analytical strategies.

**TABLE 5:** Methodological quality (JBI) and certainty of evidence (GRADE) among included studies.

Reference	Risk of bias (JBI)	Certainty of evidence (GRADE)
García-Cardoso et al. (2024)	High risk	Very low
Pallotti et al. (2024)	High risk	Very low
Zhuravleva et al. (2021)	Moderate risk	Low
Rocca et al. (2020)	High risk	Very low
Arda et al. (2019)	Moderate risk	Low
Brites-Anselmi et al. (2019)	Moderate risk	Low
Segura et al. (2019)	Moderate to high risk	Low
Mostaza et al. (2018)	Moderate risk	Low
Lacchini et al. (2018)	Moderate risk	Low
Azevedo et al. (2017)	Moderate risk	Low
Yang et al. (2017)	Moderate risk	Low
Lee et al. (2017)	Moderate risk	Low
Marchal-Escalona et al. (2016)	Moderate risk	Low
Liu et al. (2015)	Moderate risk	Low
Lacchini et al. (2013)	Moderate risk	Low
Muniz et al. (2013)	Moderate risk	Low
Hermans et al. (2012)	Moderate risk	Low
Safarinejad et al. (2011)	Low risk	Low
Andersen et al. (2011)	Moderate risk	Low
Safarinejad et al. (2010)	Moderate risk	Low
Sinici et al. (2010)	Moderate risk	Low
Lombardo et al. (2010)	Moderate risk	Low
Andersen et al. (2010b)	Moderate risk	Low
Eisenhardt et al. (2010)	Moderate risk	Low
Andersen et al. (2010a)	Moderate risk	Low
Meluzín et al. (2009)	Moderate risk	Low
Erol et al. (2009)	Low risk	Low
Lee et al. (2009)	Low risk	Low
Mazo et al. (2008)	Moderate risk	Very low
Lee et al. (2008)	Low risk	Low
Lee et al. (2007)	Low risk	Low
Peskircioglu et al. (2007)	Moderate risk	Very low
Eisenhardt et al. (2003)	Moderate risk	Very low
Liu et al. (2024)	Moderate risk	Moderate
Perticarrara Ferezin et al. (2023)	Moderate risk	Low

Source: Prepared by the author.

## 4 Discussion

Erectile dysfunction (ED) is increasingly recognized as a multifactorial condition that may be modulated by genetic factors, resulting from the complex interactions among endothelial, neurovascular, and metabolic pathways. The present systematic review synthesized evidence from 36 studies investigating genetic and pharmacogenetic biomarkers associated with ED risk, severity, and therapeutic response.

Collectively, these studies indicate that most of the genetic investigations to date have focused on the nitric oxide (NO) signaling cascade and its related regulatory genes, *NOS1*, *NOS3, PDE5A, PDE11A, VEGF, ARG1/2 and DDAH1/2*, besides *ACE* (renin-angiotensin-aldosterone system) and *MTHFR* (hyperhomocysteinemia related gene), highlight the biological relevance role of vascular-endothelial integrity and NO bioavailability in erectile physiology. Despite this convergence, the overall body of evidence remains heterogeneous and at times inconsistent, reflecting methodological limitations and population-specific variability.

### 4.1 Nitric oxide synthase (NOS) polymorphisms and ED susceptibility

Among all genetic markers examined, *NOS3* (endothelial nitric oxide synthase, eNOS) variants have been the most extensively studied. The G^894^T (Glu298Asp), T^-786^C, and intron 4 VNTR polymorphisms have been frequently associated with impaired endothelial NO synthesis and increased ED risk in multiple case–control studies across Iran, Turkey, and East Asia (Safarinejad et al., 2010; Sinici et al., 2010; Lee et al., 2009; Lee et al., 2007; Erol et al., 2009). The Iranian cohort (Safarinejad et al., 2010), with strict exclusion of metabolic and cardiovascular comorbidities, reported strong independent associations between the T^-786^C (CC) and G^894^T (TT) genotypes and vasculogenic ED (adjusted OR 4.67, p < 0.001). Similarly, Turkish and Taiwanese studies reported that carriers of the T^-786^C C allele and G^894^T T allele tended to present higher ED prevalence and lower IIEF scores, which is biologically consistent with reduced eNOS activity and diminished endothelial NO release (Sinici et al., 2010; Lee et al., 2009; Lee et al., 2007).

Conversely, large cross-sectional studies from Brazil and Europe (Andersen et al., 2010a; Meluzín et al., 2009; Eisenhardt et al., 2003) did not detect statistically significant associations, particularly in populations with high cardiometabolic burden, suggesting that gene–environment interactions, ethnic background, and comorbidity clustering may mask genetic effects. In diabetic cohorts, however, specific *NOS3* variants—such as G^894^T TT—were associated with markedly higher ED prevalence (Hermans et al., 2012), supporting a potential interaction between NO pathway polymorphisms and metabolic endothelial injury.

More recently, neuronal NOS (*NOS1*) variants have been explored in relation to the neurogenic component of ED. The Brazilian study (Perticarrara Ferezin et al., 2023) reported an association between the rs2682826 polymorphism was associated with worse erectile function scores, suggesting a possible neuronal contribution through impaired NO release from cavernous nerve terminals. Taken together, these findings suggest that disruption of the NO pathway—both endothelial and neuronal—may represent a relevant molecular axis in ED pathophysiology, although the overall certainty of evidence remains limited.

### 4.2 Phosphodiesterase genes and pharmacogenetic modulation

The main consequence of stimulated nitric oxide signaling in smooth muscle cells is the increase in cyclic guanosine monophosphate (cGMP) levels. In line with this mechanism, the main drug used in ED pharmacological treatment (i.e., Sildenafil) aims to increase cGMP levels by inhibiting its degradation by phodiesterases (PDEs). Variations in *PDE5A* and PDE11A have been investigated in relation to baseline erectile capacity and responsiveness to PDE5 inhibitors. In a well-characterized Spanish cohort, PDE5A rs3806808 G allele carriers were reported to exhibit poorer sildenafil response, particularly among non-diabetic men (Marchal-Escalona et al., 2016), suggesting a possible attenuation of cGMP accumulation and smooth muscle relaxation. A complementary investigation (García-Cardoso et al., 2024) reported that persistent post-prostatectomy ED was accompanied by upregulated *PDE5* expression in buccal mucosa, although no clear genotype–phenotype association for rs3806808 was identified, indicating that post-surgical endothelial remodeling may outweigh genetic predisposition.

Polymorphisms in *PDE11A* and *CYP2D7*, involved in cyclic nucleotide hydrolysis and sildenafil metabolism, have also been associated with variability in treatment response and adverse effects (Rocca et al., 2020). Specifically, *PDE11A* rs10201180 and *CYP2D7* rs56127449 were associated with better therapeutic response, while *PDE2A/PDE3A* variants were associated with adverse reactions. These exploratory pharmacogenomic results support a potential role for personalized therapy in ED management. Collectively, these findings are consistent with prior evidence (Lacchini and Tanus-Santos, 2014; Azevedo et al., 2017) suggesting that genetic variation within the NO–cGMP pathway may influence pharmacological efficacy of PDE5 inhibitors.

### 4.3 VEGF and angiogenic modulation of erectile function

The vascular endothelial growth factor (*VEGF*) gene is involved in penile hemodynamics by promoting angiogenesis and maintaining endothelial NO synthase expression, while at the same time, being essential to nerve recovery after nerve sparing radical prostatectomy (Ghavam et al., 2025). Multiple studies within this review reported associations between *VEGF* promoter polymorphisms—notably C^−2578^A, G^−1154^A, and G^−634^C influence both ED risk and PDE5I responsiveness. In the seminal work (Lacchini et al., 2013), the −1154 AA genotype was more frequently observed among poor responders to sildenafil in both clinical and post-prostatectomy ED, while the −2578A allele was associated with increased risk of therapeutic non-response. These findings were subsequently observed in a large Taiwanese population study (Liu et al., 2015), which described a dose-dependent relationship between the A allele of VEGF −2578C>A and both ED prevalence and metabolic syndrome, suggesting a link between angiogenic dysfunction to systemic endothelial pathology.

Taken together, this body of evidence suggests that *VEGF* genotyping may be informative within multi-marker panels that assess both vascular health and drug response. Mechanistically, *VEGF* polymorphisms may influence the NO–cGMP cascade through altered endothelial regeneration and capillary density, thereby contributing to variability in erectile function and pharmacological sensitivity, and may also be involved in neuronal function after nerve injury related ED.

### 4.4 *ACE, ARG, DDAH*, and other endothelial regulatory genes

Beyond the canonical NO and VEGF pathways, several genes regulating vascular tone and arginine metabolism have been investigated in relation to ED susceptibility and treatment outcomes. ACE enzyme produces Angiotensin II, a potent vasoconstrictor that counters the vasorelaxant effects of nitric oxide. Besides the direct effect, Angiotensin II may increase superoxide formation, which promptly reacts with NO to form peroxynitrite, a deleterious molecule involved in nitrosative damage related to chronic cardiovascular disease. Moreover, physiological increases in angiotensin II following orgasm contribute to penile detumescence, and it has been hypothesized that sustained or exaggerated activation of this pathway may impair erectile function. The *ACE* I/D polymorphism has been associated with heterogeneous findings. While some population-based studies (Safarinejad et al., 2010; Andersen et al., 2011) reported no significant association, one study (Mazo et al., 2008) reported that the DD genotype was associated with more severe ED in men with metabolic syndrome, suggesting a possible interaction between renin–angiotensin system and reduced NO availability. Similarly, Brazilian population data (Andersen et al., 2010a) described an age-dependent association of the I allele, conferring higher ED risk among men aged 40–55 years, potentially mediated by altered ACE expression or interaction with eNOS variants.

Genes regulating arginine metabolism, such as *ARG1, ARG2*, and *DDAH1/2*, have also been examined as potential functional modifiers. One study (Lacchini and Tanus-Santos, 2014) reported associations between *ARG1* and *ARG2* polymorphisms influenced plasma arginase activity and sildenafil responsiveness, while another study (Azevedo et al., 2017) found that *DDAH2* rs805304/rs805305 variants were associated with improved response to sildenafil in post-prostatectomy ED, possibly through modulation of ADMA (asymmetric dimethylarginine) and NO availability. In parallel, it has been reported (Brites-Anselmi et al., 2019) that *DDAH1/2* variants were associated with differences in ADMA and nitrite concentrations specifically among men with ED, suggesting genotype-dependent metabolic susceptibility.

Collectively, these findings support the concept that alterations in L-arginine–NO metabolism may represent an import molecular substrate of ED, in which the balance between NO synthesis (*NOS3*), substrate availability (*ARG/DDAH*), and downstream second messenger availability (*PDE5A*) may contribute to interindividual variability in disease susceptibility and treatment responsiveness.

### 4.5 One-carbon metabolism and emerging genetic markers

Polymorphisms in *MTHFR*, a key enzyme in folate-dependent homocysteine metabolism, have been investigated in relation to endothelial dysfunction and early-onset vasculogenic ED. A study (Safarinejad et al., 2010) reported that the C^677^T TT genotype was associated with early-onset ED (OR 3.14, p = 0.002), in the context of hyperhomocysteinemia and reduced folate levels. The clinical relevance of this observation was further explored by another study (Lombardo et al., 2010), which reported that supplementation with folic acid and vitamin B6 was associated with improved PDE5I responsiveness in patients with the TT genotype and elevated homocysteine. These results suggest the presence of a potentially modifiable metabolic pathway where gene–nutrient interactions may influence endothelial function and pharmacological efficacy.

Additional candidate genes such as androgen receptor (*AR*) CAG repeat polymorphisms (Liu et al., 2024; Pallotti et al., 2024) and *GNB3* C825T (Lee et al., 2008) have also been evaluated, though their associations with ED have been weak or inconsistent. The androgen receptor (AR) is primarily activated by testosterone, which in turn stimulates the eNOS–NO–cGMP signaling pathway, thereby contributing to relaxation of the smooth muscle of the corpus cavernosum (Bhasin and Snyder, 2025). Conversely, β-adrenergic signaling in penile erectile function is mediated by its associated G protein complex, which may include β subunits encoded by GNB3 (Han et al., 2023). Activation of the β3-adrenergic receptor in the human corpus cavernosum has been shown to induce vasorelaxation through a cGMP-dependent but nitric oxide–independent pathway, involving hydrogen sulfide signaling and inhibition of the RhoA/Rho-kinase pathway (Mitidieri et al., 2017). Taken together, these markers may exert indirect effects through hormonal or vascular intermediates rather than direct modulation of erectile physiology.

### 4.6 Methodological quality and evidence certainty

Although collectively informative, the methodological rigor of the included studies remains limited. Approximately three-quarters of the studies were classified as having moderate risk of bias, and nearly all were rated as low or very low certainty according to the GRADE framework. The most common methodological limitations included small sample sizes (median <150 participants), lack of replication cohorts, and inconsistent control of confounding variables such as age, diabetes, hypertension, dyslipidemia, and hypogonadism. Many studies relied exclusively on self-reported IIEF questionnaires without objective vascular assessments (e.g., penile Doppler or flow-mediated dilation).

Moreover, Hardy–Weinberg equilibrium was not verified in a significant proportion of studies, and several failed to correct for multiple comparisons. Given the substantial heterogeneity in ancestry, phenotyping strategies, and study design, formal meta-analysis and heterogeneity testing were not feasible, and a narrative synthesis was therefore adopted to contextualize findings within their respective populations rather than infer pooled effect estimates. These factors substantially limit statistical robustness and likely contribute to the inconsistency observed across studies. In addition, formal assessment of publication bias (e.g., funnel plots or statistical tests) was not feasible due to the absence of quantitative synthesis and meta-analysis, further limiting the ability to evaluate small-study effects.

Within this methodological context, the available evidence suggests that genetic variability within NO-related and vascular regulatory genes may be associated with ED susceptibility and heterogeneity in pharmacological response. Rather than establishing definitive causal relationships, the convergence of findings across genes such as NOS3, DDAH, ARG, PDE5A, and VEGF points to a biologically plausible pattern centered on endothelial–NO signaling. However, although gene–environment interactions are frequently invoked as a potential explanation for inconsistent findings, the lack of formal G × E analyses in the included studies limits causal inference. Accordingly, references to G × E should be interpreted as hypothesis-generating rather than evidence-based. This pattern should be interpreted cautiously, as the overall certainty of evidence remains low and the reported associations require validation in larger, well-designed, and replicated studies.

### 4.7 Limitations

This systematic review has several limitations. Despite a comprehensive search strategy, publication bias and language restrictions may have contributed to the omission of relevant studies. The heterogeneity of study designs, small sample sizes, and low methodological quality of many included articles substantially limited the possibility of quantitative synthesis and contributed to the low certainty of evidence. In addition, the included studies were conducted in ethnically diverse populations with substantial differences in allele frequencies for key variants (e.g., NOS3 G894T), which limits direct cross-population comparability. Moreover, the absence of subgroup analyses by ethnicity or comorbidities and the lack of ancestry-stratified analyses may have masked population-specific genetic effects or introduced ecological bias. It is also possible that the analysis of haplotypes or diplotypes combining different markers within a gene could provide more informative genetic insights, which were not consistently addressed in the included studies. Finally, as this review relied solely on published data, the presence of unpublished negative results cannot be ruled out, potentially inflating the strength of reported associations.

An additional limitation relates to phenotypic heterogeneity. Most included studies relied predominantly on the IIEF questionnaire as the sole diagnostic criterion, without objective differentiation of ED subtypes (e.g., vasculogenic, neurogenic, psychogenic). This limited phenotypic resolution may lead to misclassification and dilution of genetic associations, particularly for variants with subtype-specific effects, such as NOS3 polymorphisms that appear more consistently associated with purely vasculogenic ED. When combined with population heterogeneity, this phenotypic ambiguity further complicates the interpretation of genetic signals across studies.

Another important limitation is the systematic absence of formal gene–environment interaction (G × E) analyses. Most included studies evaluated genetic main effects without testing interaction terms between genetic variants and major environmental or clinical modifiers (e.g., metabolic syndrome, diabetes, obesity, smoking). In a multifactorial condition such as erectile dysfunction, this omission may bias genetic effect estimates and contribute to heterogeneous or attenuated associations across studies.

An additional limitation is the absence of large-scale genome-wide association studies (GWAS), as most available evidence is derived from candidate-gene approaches, limiting the assessment of the polygenic architecture of erectile dysfunction and the generalizability of genetic associations. Finally, the translational relevance of the identified genetic associations is constrained by their modest population-attributable risk. Compared with established cardiometabolic risk factors, such as diabetes or smoking, most genetic variants confer limited impact at the population level, suggesting that indiscriminate genetic testing is unlikely to be cost-effective, particularly in resource-limited healthcare systems. However, as increasingly genomic data may be available, it is possible to apply genomic data generated by other reasons of a given patient to personalize his ED treatment as an indirect outcome. The fact that ED usually develops within a context where hypertension, metabolic syndrome, diabetes, other endocrine dysregulations or prostate hyperplasia are present, highlights this possibility.

### 4.8 Translational and clinical implications

Clinically, understanding the genetic basis of ED has the potential to inform future management strategies, moving beyond purely empiric approaches toward more individualized frameworks. However, given the relatively low population-attributable risk of most genetic variants associated with ED, genetic testing should be viewed as complementary to, rather than a replacement for, optimization of traditional cardiovascular risk factor management. Identifying patients with unfavorable NOS3 or PDE5A genotypes may help identify individuals more likely to exhibit suboptimal response to PDE5 inhibitors, potentially informing alternative or adjunctive therapeutic strategies. Likewise, VEGF and ACE variants have been proposed as possible vascular risk indicators, which may assist clinicians in refining cardiovascular risk stratification in selected patients with ED.

From a translational perspective, an exploratory and pragmatic clinical framework can be proposed. First, comprehensive clinical, hormonal, and cardiometabolic assessment should remain the initial step in the evaluation of erectile dysfunction (Mostafaei et al., 2021). Second, genetic or pharmacogenetic testing may be considered in selected patient subgroups, such as men with early-onset ED, individuals without major cardiometabolic risk factors, patients with inadequate response to PDE5 inhibitors, or those with post-prostatectomy ED. Third, identified genetic variants should be interpreted as modifiers of disease susceptibility or treatment response, supporting clinical decision-making (e.g., explanation of non-response, anticipation of adverse effects, or refinement of vascular risk stratification), rather than as standalone diagnostic or prognostic tools. This framework is intended as an exploratory decision pathway to guide future research and clinical hypothesis generation, rather than a clinical guideline or mandatory algorithm.

Within this context, the potential clinical utility of genetic and pharmacogenetic testing appears most plausible in specific subgroups, such as men with early-onset ED, individuals without major cardiometabolic risk factors, patients with ED refractory to PDE5 inhibitors, or those with post-prostatectomy ED. Pharmacogenetic profiling may also contribute to drug safety by helping anticipate adverse reactions to PDE5 inhibitors in genetically predisposed individuals, as suggested by studies linking PDE11A and CYP2D7 variants to drug intolerance (Rocca et al., 2020). Nevertheless, the clinical applicability and cost-effectiveness of these approaches remain exploratory, and future trials integrating genetic, biochemical, and clinical predictors, as well as formal health economic evaluations, will be necessary to refine and validate these potential applications.

### 4.9 Future perspectives

Future research should aim to prioritize multiethnic, adequately powered GWAS integrating genetic, epigenetic, and proteomic data to clarify the polygenic architecture of ED. In this context, future genetic studies on erectile dysfunction should also prioritize high-resolution phenotyping, integrating objective vascular, neurogenic, and hormonal assessments. Standardized phenotyping protocols, established *a priori*, rigorous adjustment for comorbidities, and mechanistic validation (e.g., endothelial function testing, NO metabolite assays) will be essential to strengthen causal inference and avoid phenotypic misclassification.

Furthermore, future studies should incorporate ancestry-informed analytical strategies, including stratification by genetic ancestry and formal assessment of between-population heterogeneity, to disentangle shared versus population-specific genetic effects. Importantly, gene–environment interaction analyses should be explicitly incorporated as a core element of study design, with *a priori* testing of interaction terms between genetic variants and major environmental or metabolic modifiers, rather than being used as post-hoc explanatory hypotheses. The establishment of molecular and pathophysiological endotypes, rather than reliance on post-hoc subgroup analyses, may be essential to improve the detection, reproducibility, and clinical relevance of genetic associations in ED. Furthermore, integrating pharmacogenetic markers into clinical trials of PDE5 inhibitors may help evaluate personalized dosing strategies and identification of non-responders, thereby facilitating the translation of genetic findings into precision sexual medicine.

## 5 Conclusion

In conclusion, the present synthesis suggests that genetic polymorphisms of the nitric oxide and vascular signaling pathways, particularly *NOS3, VEGF, PDE5A, ARG, DDAH, ACE,* and *MTHFR* variants, may be associated with interindividual variability in ED risk and therapeutic response. Although the overall certainty of evidence is low, the available data support a biologically plausible framework in which ED can be conceptualized as a systemic vascular disorder potentially influenced by genetic predisposition. Importantly, these associations should be interpreted cautiously, and further validation in well-designed, adequately powered, and multiethnic studies is required before clinical translation into personalized management and preventive strategies within cardiovascular and metabolic precision medicine.
