# Supplementary material for: Genetic biomarkers associated with risk and therapeutic response in erectile dysfunction: a systematic review
Source: Front Pharmacol. 2026 Mar 24;17:1771865. doi: 10.3389/fphar.2026.1771865 (PMC13055537; doi:10.3389/fphar.2026.1771865)
Supplement: Supplementary file 1 [file Table1.docx]

**Supplementary Table S1.** Full-text articles excluded after eligibility assessment and reasons for exclusion.

| **Reference** | **Title** | **Reason for exclusion** |
| --- | --- | --- |
| Ors et al. | Erectile dysfunction in patients with cardiovascular disease and the potential influence of genetic markers | Not available in full and Triplicate article |
| Khripun et al. | Androgen receptor gene polymorphism modulates symptoms of androgen deficiency and erectile function in men with type 2 diabetes and hypogonadism | Virtual congress summary |
| Arda et al. | The association of Intron 4 VNTR and Glu298Asp polymorphisms of the nitric oxide synthetase 3 gene and vasculogenic erectile dysfunction in Turkish men | Duplicate article |
| Gonzalez-Cadavid et al. | MicroRNAs as Biomarkers of Stem Cell Damage Affecting Autologous Stem Cell Therapy for Erectile Dysfunction and Other Conditions | Not available in full |
| Jorgenson et al. | Erectile dysfunction genomics (edge): A gwas of erectile dysfunction in the kaiser permanente of northern california (KPNC) genetic epidemiology research on aging (GERA) cohort | Not available in full |
| Gamidov et al. | The role of angiotensin-converting enzyme gene polymorphism in development of erectile dysfunction in patients with metabolic syndrome | Article in Russian and not available in full |
| Erkan et al. | Polymorphism of endothelial nitric oxide synthase gene in patients with erectile dysfunction | Not available in full |
| Salvi et al. | Cardiovascular effects of sildenafil in hypertensive men with erectile dysfunction and different alleles of the type 5 cGMP-specific phosphodiesterase (PDE5) | Inadequate population |
| Gao et al. | The association between serum 25-hydroxyvitamin D levels and erectile dysfunction: a two-sample Mendelian randomization analysis | Ineligible study type |
| Meng et al. | Comprehensive analysis of biological landscape of oxidative stress-related genes in diabetic erectile dysfunction | Inadequate population |
| Wang et al. | Correction: Integrated Mendelian randomization and single-cell RNA-sequencing analyses identified OAS1 as a novel therapeutic target for erectile dysfunction via targeting fibroblasts | Correction/Errata |
| Fang et al. | Increased walking pace reduces the rate of erectile dysfunction: results from a multivariable Mendelian randomization study | Ineligible study type |
| No authors listed | Correction to: No bidirectional association between serum 25-hydroxyvitamin D and erectile dysfunction: Mendelian randomization and genetic association studies | Correction/Errata |
| Safarinejad et al. | Retraction notice to "G-protein ß3 subunit gene 825C/T polymorphism and its association with the presence, severity, and duration of vasculogenic erectile dysfunction" | Correction/Errata |
| Zhang et al. | The impact of cannabis use on erectile dysfunction and sex hormones: a Mendelian randomization analysis | Ineligible study type |
| Woolf et al. | A drug target for erectile dysfunction to help improve fertility, sexual activity, and wellbeing: mendelian randomisation study | Ineligible study type |
| Chen et al. | Association between atorvastatin and erectile dysfunction: a comprehensive analysis incorporating real-world pharmacovigilance and Mendelian randomization | Ineligible study type |
| Zhang et al. | The indicative effects of apolipoproteins on organic erectile dysfunction: bridging Mendelian randomization and case-control study | Ineligible study type |
| Wensong et al. | Mendelian Randomization Reveals Serum Copper as a Micronutrient is a Risk Factor for Erectile Dysfunction | Ineligible study type |
| Zhu et al. | Mitochondrial FIS1 As a Novel Drug Target for the Treatment of Erectile Dysfunction: A Multi-Omic and Epigenomic Association Study. | Ineligible study type |
| Liu et al. | Multi-omics association study integrating GWAS and pQTL data revealed MIP-1α as a potential drug target for erectile dysfunction | Ineligible study type and Duplicate article |
| An et al. | Glucagon-like peptide-1 receptor agonists and the risk of erectile dysfunction: a drug target Mendelian randomization study | Ineligible study type |
| Deng et al. | Branched-chain amino acids levels associated with risk of erectile dysfunction: A Mendelian randomization analysis | Not available in full |
| Zhang et al. | Exploring the causal associations of gout and serum uric acid levels on erectile dysfunction: A Mendelian randomization study | Ineligible study type |
| Zhang et al. | Causal effects of gut microbiota on erectile dysfunction: a two-sample Mendelian randomization study | Ineligible study design |
| Sun et al. | Causal associations between erectile dysfunction and high blood pressure, negative psychology: a Mendelian randomization study | Ineligible study design |
| Zhu et al. | Association of high LDL concentrations with erectile dysfunction from a Mendelian randomization study | Ineligible study design |
| Sun et al. | Serum 25-hydroxyvitamin D level and erectile dysfunction: a causal relationship? Findings from a two-sample Mendelian randomization study | Ineligible study type |
| Chen et al. | Causal relationship between gut microbiota and male erectile dysfunction: a Mendelian randomization analysis | Ineligible study type and Duplicate article |
| Qiu et al. | Exploring novel drug targets for erectile dysfunction through plasma proteome with genome | Ineligible study type |
| Çam et al. | Association of angiotensin-converting enzyme I/D and eNOS G894T gene polymorphisms with erectile dysfunction | Not available in full |
| Kim et al. | Determination of human angiotensin converting enzyme (ACE) gene polymorphisms in erectile dysfunction: frequency differences of ACE gene polymorphisms according to the method of analysis | Not available in full |
| Perlis et al. | Genetic and clinical predictors of sexual dysfunction in citalopram-treated depressed patients | Inadequate population |
| Chen et al. | Identification of Systemic Drug Targets for Anti-cavernous Fibrosis in the Treatment of Erectile Dysfunction, Guided by Genome-Wide Mendelian Randomization | Ineligible study type |
| Wang et al. | Mendelian randomization analyses reveal causal relationships between chronic psychological stress and risk of erectile dysfunction | Ineligible study type |

Note: One article was identified in triplicate and two articles were duplicated, resulting in a total of 35 excluded records.
